# Kidney biopsy in patients with glomerulonephritis: is the earlier the better?

**DOI:** 10.1186/1471-2369-13-34

**Published:** 2012-06-08

**Authors:** Dominik G Haider, Alexander Friedl, Slobodan Peric, Günther F Wiesinger, Michael Wolzt, Julian Prosenz, Henrik Fischer, Walter H Hörl, Afschin Soleiman, Valentin Fuhrmann

**Affiliations:** 1 Department of Nephrology and Dialysis, University Hospital Vienna, Medical University of Vienna, Waehringer Guertel 18-20, 1090, Vienna, Austria; 2 Department of Physical Medicine and Rehabilitation, University Hospital Salzburg, Paracelsus Medical University, Müllner Hauptstrasse48, 5020, Salzburg, Austria; 3 Department of Clinical Pharmacology, University Hospital Vienna, Medical University of Vienna, Waehringer Guertel 18-20, 1090, Vienna, Austria; 4 Department of Anesthesiology, University Hospital Vienna, Medical University of Vienna, Waehringer Guertel 18-20, 1090, Vienna, Austria; 5 Department of Clinical Pathology, University Hospital Vienna, Medical University of Vienna, Waehringer Guertel 18-20, 1090, Vienna, Austria; 6 Department of Gastroenterology, University Hospital Vienna, Medical University of Vienna, Waehringer Guertel 18-20, 1090, Vienna, Austria

## Abstract

**Background:**

Interventional diagnostic procedures are established for several diseases in medicine. Despite the KDOQI guideline recommendation for histological diagnosis of kidney disease to enable risk stratification, its optimal time point has not been evaluated. We have therefore analyzed whether histological diagnosis of glomerulonephritis (GN) at an early stage of chronic kidney disease (CKD) is associated with different outcome compared to diagnosis at a more advanced stage.

**Methods:**

A cohort of 424 consecutive patients with histological diagnosis of GN were included in a retrospective data analysis. Kidney function was assessed by glomerular filtration rate (GFR) estimation at the time point of kidney biopsy and after consecutive immunosuppressive therapy. Censored events were death, initiation of dialysis or kidney transplantation, or progression of disease, defined as deterioration of CKD stage ≥1 from kidney biopsy to last available kidney function measurement.

**Results:**

Occurrence of death, dialysis/transplantation or progression of disease were associated with GFR and CKD stage at the time of kidney biopsy (*p* < 0.001 for all). Patients with CKD stage 1 and 2 at kidney biopsy had fewer endpoints compared to patients with a GFR of <60 ml/min (*p* < 0.001).

**Conclusion:**

Kidney function at the time point of histological GN diagnosis is associated with clinical outcome, likely due to early initiation of specific drug treatment. This suggests that selection of therapy yields greatest benefit before renal function is impaired in GN.

## Background

The time points when interventions should be recommended have been investigated and established in multiple fields of medicine. For patients with renal disease kidney biopsy can be necessary for diagnosis and treatment selection. Technical advances (e.g. real-time ultrasound and automated biopsy needles) improved the general implementation of this procedure [1]. Major complications occur in less than <0.1% of kidney biopsies [1-4].

The incidence of chronic kidney disease (CKD) is increasing in the western communities and outcomes are poor. The number of patients with CKD undergoing maintenance hemodialysis is expected to rise further [5]. Complications of decreased kidney function or concomitant cardiovascular disease (CVD) may be preventable by early diagnosis and treatment [6]. However, CKD is frequently underdiagnosed and undertreated [7-9].

There is insufficient evidence to conclude best timing of invasive diagnostic procedures in patients with several kidney diseases. An early kidney biopsy may be of particular interest for those patients where disease classification is based on histological diagnosis and where the disease progression can be mitigated by treatment. While current guidelines have established the medical management of glomerulonephritis (GN) including the necessity of histological analysis and administration of immunosuppressive therapies, it is yet unclear if early diagnosis may result in prevention of CKD development in this heterogeneous group of patients.

We therefore performed a retrospective analysis to address the question if diagnosis of GN from an early kidney biopsy before kidney function impairment, as classified by glomerular filtration rate (GFR) and CKD stage, is associated with different outcome compared to diagnosis of GN at a later CKD stage.

## Methods

**Figure 1:**
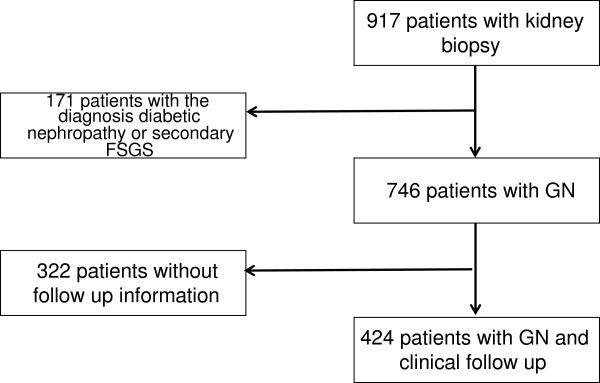
Subject identification chart.

Retrospective data from 917 consecutive patients who underwent kidney biopsy between 1992 and 2009 were identified. Among those 171 patients had diabetic nephropathy or secondary focal segmental glomerulosclerosis (FSGS) and were excluded. From 322 patients no follow up was available. Thus, 424 patients with a histological diagnosis of GN who received immunosuppressive therapy were included into the analysis (Figure 1). The study protocol and data handling procedure was approved by the Ethics Committee of the Medical University of Vienna.

Patients were characterized by CKD-stages according to their kidney function prior to biopsy and during follow up. Glomerular filtration rate (GFR) estimation was calculated via the MDRD formula [10]. Primary outcome variables were progression of disease, dialysis/kidney transplantation or death. Progression of kidney disease was defined as deterioration of CKD stage ≥1 from the time of kidney biopsy to last kidney function measurement. Stable disease was regarded as constant or improved CKD stage from the time of kidney biopsy to last available kidney function measurement, without an episode of dialysis or kidney transplantation. After kidney biopsy patients received induction therapy with cortisone with or without cyclophosphamide as immunosuppressive therapy according to their histological diagnosis and adjusted to their body weight. Systolic blood pressure was set at ≤130 mmHG. The amount of patients with the respective antihypertensive therapy: ACE-inhibitors/AT2-blockers: 190/96 (withdrawal because of systolic BP < 110 mmHg); ß-Blocker: 77; Ca-channel-blockers:106; alpha-blockers:53.

Indications for kidney biopsy were proteinuria, microhematuria or increments in serum creatinine alone or in combination. The histological classification of GN was derived from laboratory reports of the Department of Pathology at the Vienna General Hospital, which has also been used for clinical decision making in these patients. Baseline characteristics and co-morbidities are shown in Table 1.

**Table 1:** **Baseline characteristics and co-morbidities in patients with GN (*n*** **= 424)**

Baseline characteristics	
Age	54 (39;67)
Male/female	240/185
IgA-Nephropathy	65
Primary Focal Segmental Glomerulosclerosis	46
Minimal Change GN	37
Membranous GN	122
Membranoproliferative GN	39
Vasculitis	57
Systemic Lupus Erythematosus	51
Goodpasture Syndrome	7
Co-Morbidities	
Diabetes Mellitus Type 1	4
Diabetes Mellitus Type 2	27
Hypertension	237
Coronary Heart Disease	28
Peripheral Vascular Disease	14
Stroke	8
Thrombembolic Complication	33
Hyperlipidemia	85
Malignancy	22
Chronic Obstructive Pulmonary Disease	27

### Statistical analysis

For data description results are presented as median and 25–75% interquartile range or mean and standard deviation as appropriate. For univariate analysis the Mann Whitney *U* test or the chi-squared test were used as appropriate. The Kaplan-Meier method was used to determine event-free survival and the log rank test was used to compare survival between subgroups. Univariate and multivariate regression analysis were performed using the Cox proportional hazard regression model to determine the effect of various variables on survival. Potential predictors were defined a priori or based on associations in the univariate analysis at a conservative threshold (*p* < 0.10). The Hosmer-Lemeshow test was used to assess goodness of model fit. We used SPSS Statistics for data management and calculations. A two sided *p*-value less than 0.05 was considered statistically significant. The authors performing the data analysis (D.G.H. and V.F.) were masked and were not involved in the data acquisition.

## Results

The median observation time was 4.0 years (interquartile range, IQR: 1.3;8.4 years). Overall, 106 (25%) patients had CKD stage 1, 65 (15%) patients had CKD stage 2, 109 (26%) patients had CKD stage 3, 70 (17%) patients had CKD stage 4, and 74 (17%) patients had CKD stage 5 before kidney biopsy. Estimated GFR slightly improved in pooled analysis during the observation period from 45 ml/min (IQR: 23–80) to 51 ml/min (IQR: 25–83) after 1 month and to 52 ml/min (IQR: 29–76) at the end of the observation period (*P* < 0.05 vs baseline). In subgroup analysis significant improvements in GFR were seen after one month for patients with vasculitis, IgA-Nephropathy and Systemic Lupus Erythematosus (SLE) (all *p* < 0.05), and at study end for patients with membranous GN, IgA-Nephropathy and vasculitis (all *p* < 0.05).

**Figure 2:**
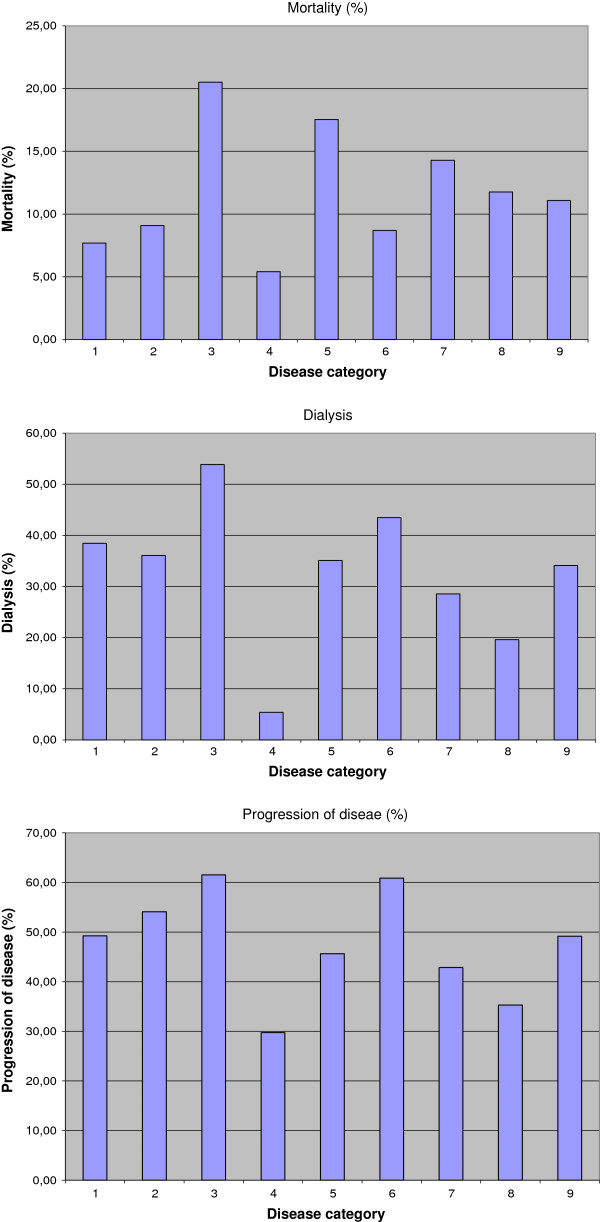
Distribution of mortality, dialysis/transplantation and progression of disease according to diagnosis and overall.

Kidney function remained stable or improved in 216 (51%) patients. 208 patients had a progression of disease (49%). Of these, 144 patients underwent dialysis (34%) and 98 patients had subsequent kidney transplantation (23%). 47 patients (11%) died during the observation period. Distribution of mortality, dialysis/transplantation and progression of disease according to histological diagnosis is shown in Figure 2.

Data of quantitative proteinuria one month after initiation of immunosuppressive therapy was accessible in 174 patients. Of these 42 patients (24%) had proteinuria of >3.5 g/d. Univariate regression analysis including underlying diseases, age, sex and GFR identified membranous GN and vasculitis as predictors for progression/persistence of proteinuria (for both *p* < 0.01). Multivariate regression analysis detected membranous GN as an independent predictor for progression/persistence of proteinuria (*p* < 0.01).

**Figure 3:**
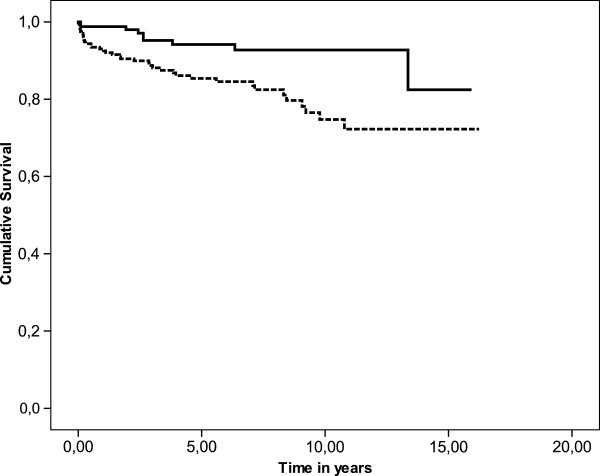
**Cumulative survival of patients with CKD stages 1 + 2 (solid line, n = 172) compared with patients with CKD stages 3, 4 and 5 (dotted line, n = 248) (Kaplan Meier,*p*** **= 0.001; Chi-Square-Test).**

**Figure 4:**
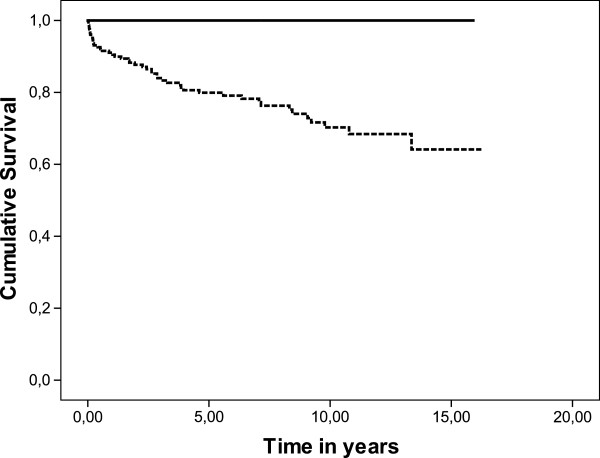
**Cumulative survival of patients with stable (solid line, n = 209) or progressive kidney disease (dotted line, n = 215) (Log Rank test*p*** **< 0.0001).** Progression of kidney disease was defined as deterioration of CKD stage ≥1 from the time of kidney biopsy to last kidney function measurement.

To assess whether the time point of kidney biopsy is associated with survival, patients with a GFR ≥60 ml/ml (CKD stage 1 and 2; *n* = 171) were compared with the group of subjects with impaired renal function at CKD stages 3, 4 and 5 (*n* = 253). Patients undergoing kidney biopsy at CKD stage 1 and 2 had a markedly better overall event-free survival than the group of patients with higher CKD stages (Figure 3). Dialysis or kidney transplantation was less frequent in patients with CKD stage 1 and 2 after immunosuppressive therapy than other patients with GN (*p* < 0.01). Disease progression was associated with increased mortality during follow-up (Figure 4).

**Figure 5:**
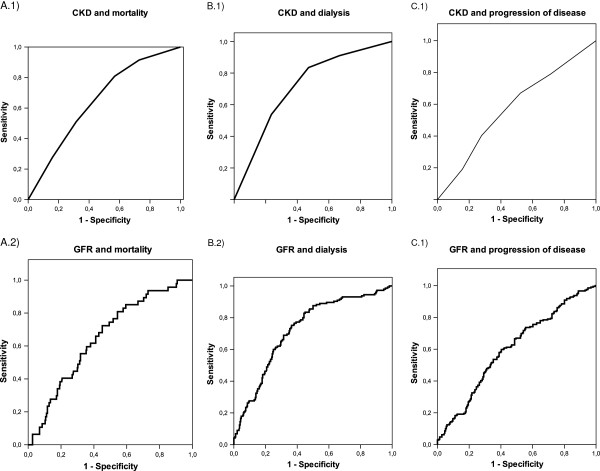
ROC-Analysis for CKD and GFR at the time point of kidney biopsy as predictors for mortality (A), dialysis (B) and progression of disease (C).

CKD stage at kidney biopsy was associated with favorable renal outcome. In particular, CKD stage was predictive for mortality (AUC: 0.65 for both), dialysis/transplantation (AUC: 0.71 and 0.73), and progression of disease (AUC: 0.58 and 0.6) (Figure 5).

Multivariate regression analysis revealed that mortality was associated with CKD stages, but not with dialysis/transplantation and progression of disease (Table 2). Independent predictors for dialysis/transplantation were CKD stage and minimal change GN in multivariate regression analysis (Table 2). Minimal change GN was the only predictor for progression of disease in a multivariate model (Table 2). Further, mortality was associated with age and MP-GN.

**Table 2:** Multivariate Cox-Regression for mortality (A), for dialysis/transplantation (B) and for progression of chronic kidney disease (C)

	***p*-value**
A)	
CKD stage	0.047
Age	0.001
Vasculitis	0.396
Membranoproliferative GN	0.02
B)	
CKD stage	<0.001
Systemic Lupus Erythematosus	0.173
Minimal Change GN	0.02
Membranoproliferative GN	0.06
C)	
CKD stage	0.19
Age	0.09
Minimal Change GN	0.04
Systemic Lupus Erythematosus	0.09

## Discussion

In this large sample of patients with different etiology of GN an early time point of kidney biopsy was associated with smaller declines in kidney function, a less frequent incidence of dialysis/transplantation, and death. Preserved kidney function at the time point of biopsy was a strong predictor of salutary outcome in patients with CKD.

Overall, immunotherapy prevented deterioration of kidney function in approximately half of the cohort of patients with GN. This treatment response is comparable with that shown across the groups of subjects with different histological diagnoses of GN in other studies [11-17]. When patients were in CKD stage 1 or 2 progression of disease was less likely during therapy indicating that early specific drug treatment is essential for clinical benefit, independent of the underlying classification of GN. However, this effect was not consistent and the patients with membranoproliferative GN had the worse outcome. This is in accordance with other data [18].

The association of CKD stages with mortality is comparable with data obtained in the general population [18,19]. Our data demonstrates that early biopsy-guided initiation of therapy might result in a profound advantage for patients to preserve kidney function. Patients at CKD stage 1 and 2 had a significantly better outcome compared with other patients. This is also in good agreement with studies in patients with high cardiovascular risk [20,21].

Our analysis revealed strong associations of mortality with age. This is in accordance with other data regarding the progression of disease [22]. Kidney biopsy has demonstrated a clear benefit also for elderly patients with acute kidney failure in an observational study [23]. These findings are extended by our results in patients with GN. Therefore, kidney biopsy should be considered independent of age in all patients with suspected GN. Current indications for kidney biopsy are derived from surveys and expert opinions [24,25]. This study adds important evidence based on a large cohort analysis.

A limitation of the analysis of efficiency of drug treatment is the fact that response rates have improved over time by the availability of refined dosing schemes and new drugs. However, this does not limit the principal finding of this observational study, because no therapy was withheld and all patients had access to optimized medical treatment provided by the national sickness fund during the observation period. This study has not compared individual treatments within groups of patients with different GN entities and numbers within subgroups are too small for such analysis. While this is a large study, the median follow-up is 4 years, which may not be long enough to observe clinically relevant outcome or progression in diseases such as IgA nephropathy or membranous nephropathy. A further limitation of our study is that only a controlled interventional trial may demonstrate if baseline differences may be accounted for the results seen. However, ethical concerns prevent conduct of such a trial.

## Conclusions

In summary, kidney biopsy at CKD stages 1 or 2 and consecutive therapy preserves kidney function and prevents disease progression through early initiation of therapy. The present observational data clearly supports the concept of early kidney biopsy in patients with suspected GN.

## Competing interest

The authors declare that they have no competing interests.

## Authors’ contributions

AF, SP, HF, GFW and JP carried out the data acquisition. DGH and AS did the data administration. DGH and VF performed the statistical analysis. DGH, VF, WHH and MW wrote the manuscript. All authors read and approved the final manuscript. Authors state that they have no conflict of interest.

## Pre-publication history

The pre-publication history for this paper can be accessed here:

http://www.biomedcentral.com/1471-2369/13/34/prepub
